# NGF and proNGF Reciprocal Interference in Immunoassays: Open Questions, Criticalities, and Ways Forward

**DOI:** 10.3389/fnmol.2016.00063

**Published:** 2016-08-03

**Authors:** Francesca Malerba, Francesca Paoletti, Antonino Cattaneo

**Affiliations:** ^1^Neurotrophic Factors and Neurodegenerative Diseases Unit, European Brain Research Institute, “Rita Levi-Montalcini” FoundationRome, Italy; ^2^BioSNS Laboratory, Scuola Normale SuperiorePisa, Italy

**Keywords:** NGF, proNGF, immunoassay, ELISA, SPR, AlphaLISA, interference

## Abstract

The homeostasis between mature neurotrophin NGF and its precursor proNGF is thought to be crucial in physiology and in pathological states. Therefore, the measurement of the relative amounts of NGF and proNGF could serve as a footprint for the identification of disease states, for diagnostic purposes. Since NGF is part of proNGF, their selective identification with anti-NGF antibodies is not straightforward. Currently, many immunoassays for NGF measurement are available, while the proNGF assays are few and not validated by published information. The question arises, as to whether the commercially available assays are able to distinguish between the two forms. Also, since in biological samples the two forms coexist, are the measurements of one species affected by the presence of the other? We describe experiments addressing these questions. For the first time, NGF and proNGF were measured together and tested in different immunoassays. Unexpectedly, NGF and proNGF were found to reciprocally interfere with the experimental outcome. The interference also calls into question the widely used NGF ELISA methods, applied to biological samples where NGF and proNGF coexist. Therefore, an immunoassay, able to distinguish between the two forms is needed. We propose possible ways forward, toward the development of a selective assay. In particular, the use of the well validated anti-NGF αD11 antibody in an alphaLISA assay with optimized incubation times would be a solution to avoid the interference in the measurement of a mixed sample containing NGF and proNGF. Furthermore, we explored the possibility of measuring proNGF in a biological sample. But the available commercial kit for the detection of proNGF does not allow the measurement of proNGF in mouse brain tissues. Therefore, we validated an SPR approach for the measurement of proNGF in a biological sample. Our experiments help in understanding the technical limits in the measurement of the NGF/proNGF ratio in biological samples, and propose concrete solutions toward the solution of this problem.

## Introduction

Nerve Growth Factor (NGF) (Levi-Montalcini, 1987), the prototype neurotrophin (Bothwell, 2014), is required for the development and maintenance of neurons in the central and peripheral nervous system, as well as in non-neuronal systems, and exerts its functions *via* TrkA and p75^NTR^ receptors (Hempstead et al., 1991; Chao and Hempstead, 1995; Fahnestock et al., 2004a; Clewes et al., 2008; Paoletti et al., 2009).

NGF is translated as a precursor protein, proNGF, which is processed in the Trans-Golgi Network by furin (Shooter, 2001), and has complementary functions to its mature counterpart (Hempstead, 2006). ProNGF is the main form of NGF in the brain (Fahnestock et al., 2001; Bierl and Isaacson, 2007), and can also be secreted as such and processed by extracellular proteases (Lee et al., 2001; Bruno and Cuello, 2006). proNGF interacts mainly with p75^NTR^ and sortilin, and the interplay between these receptors triggers cellular death (Nykjaer et al., 2004; Volosin et al., 2006). It also interacts with TrkA, although with a reduced affinity (Fahnestock et al., 2004a,b).

The homeostasis between the levels of mature NGF and proNGF is emerging as a crucial factor in physiology and in many pathological states. For instance, in the adult brain, increased amounts of proNGF have been associated to neurodegeneration (Counts and Mufson, 2005), highlighting the importance of the NGF/proNGF ratio as an upstream driver for neurodegeneration (Capsoni and Cattaneo, 2006; Capsoni et al., 2010; Iulita and Cuello, 2014; Counts et al., 2016).

Therefore, the NGF/proNGF levels in cerebrospinal fluid (CSF) represents a target of clinical interest and a footprint biomarker, to be exploited for diagnostic purposes in numerous neuronal and non-neuronal pathologies (Iulita and Cuello, 2014; Counts et al., 2016).

For many years, NGF levels have been measured in biological fluids (Vizzard, 2000; Wang et al., 2010; Konukoglu et al., 2012; Liu and Kuo, 2012; Tasset et al., 2012; Counts et al., 2016) by ELISA, and a very large body of literature reports on correlations between altered levels of “*bona fide*” NGF and various pathological conditions (Vizzard, 2000; Lombardi et al., 2008; Marksteiner et al., 2008; Wang et al., 2010; Konukoglu et al., 2012; Liu and Kuo, 2012; Tasset et al., 2012; Ke et al., 2014; Camara et al., 2015), without taking into consideration the concomitant presence of proNGF in the same samples. However, the open question remains, whether ELISA assays, exploiting anti-NGF antibody pairs, really discriminate between the two neurotrophin forms. Indeed, the possibility of discriminating and measuring relative amounts of NGF and proNGF in biological samples is hampered by the fact that mature NGF is part of the proNGF protein, a simple fact that is most often overlooked.

Indeed, no evidence has been reported so far, providing a demonstration whether or not the presence of the NGF precursor is neutral to the measure of NGF, by the widely used NGF immunoassays.

Given the interest of determining the proNGF/NGF ratios in biological samples and motivated by the objective of developing a method for the selective reliable detection of the two related proteins, we set out to closely and systematically examine currently available methods. We made use of a set of anti-NGF and anti-proNGF antibodies, and validated our conclusions both with recombinant proteins and with biological samples from transgenic mice tissues. Although differences in the NGF sequences from different species exist (Malerba et al., 2015), we selected the mice samples as a validation model organism.

The different formats analyzed will be presented in this paper, which produced a set of *a priori* unexpected results, and allowed to identify major criticalities and the possible ways to overcome the obstacles.

## Methods

### Neurotrophins and proNGF transgenic mouse model

All the experiments were performed on the short forms of the recombinant mouse proNGF, namely rm-proNGF25 (according to the nomenclature reported in Paoletti et al., 2009). For the sake of brevity, throughout the manuscript the protein was simply named as proNGF. Accordingly, the mature protein was named as NGF. proNGF and NGF were expressed and purified as in Paoletti et al. (2009). The 280 nm absorbance of the purified proteins in buffer was measured by using a UV-visible spectrometer (Nanodrop). The concentrations were calculated according to the Lambert–Beer law.

The biological source for the analyzed brain tissues was the transgenic mouse TgProNGF#72 (Tiveron et al., 2013) and its WT counterpart, the BDFN strain. The homogenation of the brain extracts and the densitometric analysis were carried out as described (Tiveron et al., 2013), unless where specified differently.

Animal experiments were carried out according to Italian legislation (DL 116/92) and European Communities Council Directive (86/609/EEC). Studies were conducted under the permit (number 3/2012, EBRI Foundation) approved by the Italian National Committee for animal research.

### Immunoprecipitation and Western blot for measuring NGF and proNGF from transgenic and WT mice cortex

The immunoprecipitation (IP) and Western Blot were performed as previously described (Tiveron et al., 2013). Briefly, cerebral corteces (CTX) were isolated from WT and transgenic TgproNGF#72 mice. For each immunoprecipitation (IP), a pool of four animals, divided into male and female, was used and analyzed. Each pool of CTX was homogenized with 2 ml of Lysis buffer per g of tissue (Lysis Buffer: Tris HCl 0.1 M, NaCl 0.4 M, SDS 0.1%, Triton X-100 1%). The homogenates were incubated in ice for 30 min and then centrifuged for 30–40 min at maximum speed. The total amount of protein in the supernatant was quantified by Bradford Assay (SIGMA-Aldrich, St Louis, MO, USA). The same amount of total protein samples (4 mg) was immunoprecipitated with a large excess of anti-NGF αD11 antibody before western blot. The whole samples after IP were loaded onto the gel (Criterion, Bio-Rad, 4–12% Bis-Tris) for western blotting. The primary antibody used was anti-proNGF (Alomone) and the secondary antibody was goat anti-rabbit HRP conjugated (Jackson laboratories). Recombinant proNGF and NGF were used as control samples; loaded samples were in the linear range of detection. The membrane was then stripped and incubated with the primary antibody, anti-NGF M20 (Santa Cruz). The intensity of the bands on the western blots from three separate experiments was measured by densitometric analysis using the software of the Kodak scanner. The net intensity was normalized against the internal area of each band and then compared with an internal standard of NGF or proNGF.

### ProNGF interferes with the determination of NGF by using commercial ELISA

NGF Emax® ImmunoAssay System Promega was initially chosen to perform the experiments because it is one of the most widely used commercial kits for NGF measurement.

The assay was carried out using either the mAb Promega, as indicated in the manufacturer's protocol, or the rat anti-NGF mAb αD11 (Cattaneo et al., 1988) (0.5 μg/ml), as primary antibody. All the other steps were carried out according to manufacturer's instruction.

In order to verify the specificity of the ELISA for NGF, a curve of recombinant proNGF was carried out (10 pg/ml – 1 μg/ml range) (Supplementary Table S1). Moreover, two concentrations of NGF (10 and 100 pg/ml) and proNGF (50 and 200 pg/ml), spiked in the assay buffer, either alone or together with NGF, were assayed (Tables 1, 2 and **Figure 3**).

**Table 1:** **Recovery of NGF and proNGF spiked in the sample buffer (supplied by each supplier of the commercial kits)**.

**ProNGF/NGF spiked in sample buffer (pg/ml)**	**Recovery % of proNGF or NGF spiked**			
	**Emax Promega with mAb Promega**	**Emax Promega with mAb αD11**	**Chemikine Chemicon**	**NGF Rapid ELISA Biosensis**
proNGF 200	0	45	9	0
proNGF 50	0	28	0	0
NGF 10	66	58	139	218
NGF 10 + proNGF 50	100	266	154	241
NGF 10 + proNGF 200	115	1391	390	339
NGF 100	82	92	180	**532**
NGF 100 + proNGF 50	140	195	180	427
NGF 100 + proNGF 200	86	257	210	479

*The table synthesizes the results of the percentage of recovery of NGF and proNGF spiked separately and together into the assay buffer of the three different commercial kits for the detection of NGF analyzed. The Promega kit was tested in the two different formats: rat mAb Promega and αD11. Two concentrations of recombinant NGF (10 and 100 pg/ml) and proNGF (50 and 200 pg/ml) were spiked into the assay buffer either alone or together with NGF and measured by using the kits previously described. The percentage of recovery was calculated on the spiked value of NGF and proNGF, if spiked alone, and on the spiked value of only NGF, in the sample containing both the precursor and mature form. Description of the colored values for the recovery percentage of NGF, calculated for the mixed sample:*
– *red when the value was higher than the corresponding recovery percentage of NGF, spiked alone*; – *black if the values were similar*; – *blue if the recovery percentage of NGF, in presence of proNGF, was lower than the corresponding value of NGF spiked alone*; – *the black bold values represent the outliers values*.

**Table 2:** **NGF and proNGF spiked into brain cortex extract of proNGF#72 Transgenic Mouse assayed by Emax Promega with mAb Promega and mAb αD11**.

**ProNGF spiked into brain cortex extract of proNGF#72 Transgenic Mouse (Dil.1:200)**	**NGF Concentration obtained (pg/ml** ± **Standard deviation)**	
	**Emax Promega with mAb Promega**	**Emax Promega with mAb αD11**
0	290.04 ± 0.01	382.90 ± 0.06
proNGF 200	326.89 ± 0.03	380.24 ± 0.07
proNGF 50	351.12 ± 0.07	398.26 ± 0.03

*The table reports the values of NGF concentration obtained from the samples of proNGF#72 Transgenic Mouse cortex homogenate, spiked with 0, 50, and 200 pg/ml of recombinant proNGF. In red the outliers values. The concentration of total protein of the cortex brain extract was 27 mg/ml, then diluted 1:200*.

TgProNGF#72 brain homogenates were measured at different dilutions: 1:25, 1:50, 1:100, and 1:200. The tissue samples were homogenized as described (Tiveron et al., 2013). Cortex brain areas (CTX) were isolated from TgProNGF#72mice, 12 month old. A pool of 8 transgenic animals (4 males and 4 females) was used and analyzed. The concentration of the total protein in the sample, measured by Bradford assay (Sigma-Aldrich), was 27 mg/ml. proNGF at the same concentrations assayed in assay buffer, were spiked into TgProNGF#72 homogenate (1:25, 1:50, 1:100, and 1:200), and measured.

In order to verify the obtained results, two other commercial kits (ChemiKine Nerve Growth Factor, Sandwich ELISA, from Millipore, and Mouse NGF Rapid ELISA Kit from Biosensis) were tested. The two concentrations of NGF (10 and 100 pg/ml), and proNGF (50 and 200 pg/ml), either alone or together with NGF, were assayed spiked in the assay buffer, supplied by Promega.

**Figure 1:**
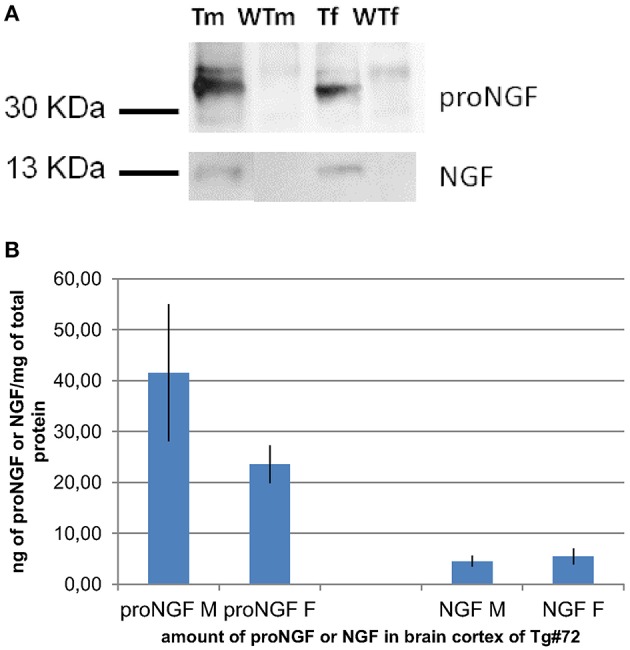
**IP and WB of transgenic and wild-type mice. (A):** IP and WB of cortex extracts from male (M) and female (F) TgProNGF#72 and wild-type (WT) mice. IP on extracts from cortex (CTX) with anti-NGF αD11 antibody, followed by WB with anti-NGF or anti-proNGF antibody, as described in Tiveron et al. (2013). A representative WB probed with anti-proNGF (PAb Alomone), (top) or anti-NGF M20 (Santa Cruz) (bottom) is shown. TgproNGF#72 and wild type mice, male and female, were analyzed. **(B)** Quantitative analysis of proNGF and mature NGF in the CTX of TgproNGF#72 mice, male and female, by IP and WB and densitometric analysis. After anti-NGF IP, the proNGF bands, (in WB probed with anti-proNGF), and the NGF bands, (in the WB probed with anti-NGF antibody), both identified also by Mass Spectrometry, were quantified. The resulting intensities were normalized against the area of the bands, and then compared with an internal standard of recombinant proNGF and NGF. Loaded samples were in the linear range of detection. Comparison between proNGF and NGF amounts in TgproNGF#72, male and female, is reported in the histogram. The experiment was carried out in triplicate.

The experiments were repeated three times for Promega kit, with both the antibodies, two times for Biosensis and Millipore kits. The obtained values were compared to IP results indicated in Figure 1B (The brain extracts used in the experiment were derived from a mixed sample of 50% male and female, therefore an average of the values, was considered).

NGF Emax® ImmunoAssay System Promega and ChemiKine Nerve Growth Factor, Sandwich ELISA Millipore were purchased, while Mouse NGF Rapid ELISA Kit Biosensis was kindly provided by Biosensis.

The statistical analysis was carried out by using a *t*-student test. The interpolated values corresponding to samples spiked with NGF and proNGF together were compared to the value of samples spiked with NGF alone, measured with the same ELISA kit.

### ProNGF interferes with the determination of NGF by using in house ELISA

In order to asses an in-house NGF ELISA, a number of different sandwich formats were tested by using both commercial antibodies and antibodies produced in our lab: Anti-NGF mAb αD11 (Cattaneo et al., 1988); Anti-NGF R&D no. MAB256; Anti-NGF pAb M20, Santa Cruz no. sc-549; Anti-NGF pAb H20, Santa Cruz no. sc-548; Anti-proNGF scFv FPro10 (Paoletti et al., 2012); Anti-proNGF pAb Sigma no. P 5498; Anti-NGF pAb Sigma no. N 6655.

The assay buffer was milk (Applichem) at a concentration of 4%.

The concentration of the antibodies in coating (mAb αD11) was 2 μg/ml in carbonate buffer. The concentration of the primary was variable. It was assessed by following the manufacturer's instructions for commercial antibodies. The Horse Radish Peroxidase (HRP) conjugated secondary antibodies were used at the concentration suggested by the manufacturer's.

The incubation times were different. For the calibration curves and the samples, it varied from 15 to 30 min, in the case of a fast kinetics, for the primary antibody it was 1.5–2 h, for the secondary antibody 1 h.

### AlphaLISA (Perkin Elmer) for the selective detection of NGF: proof of principle

AlphaLISA is a highly sensitive immunoassay, commercialized by Perkin Elmer. In the AlphaLISA assay, a biotinylated antibody, and an antibody-conjugated AlphaLISA Acceptor beads are used to capture the target analyte. The biotinylated antibody associates with an Alpha streptavidin-coated Donor bead. When the analyte is present in the sample, the Donor and Acceptor beads are brought together. Upon excitation, a photosensitizer inside the Donor bead converts ambient oxygen to an excited singlet state. Singlet oxygen diffuses up to 200 nm to produce a chemiluminescent reaction in the Acceptor bead, leading to light emission. The amount of light is proportional to the amount of analyte present in the sample.

First of all, the antibodies anti-NGF Mab αD11 (Cattaneo et al., 1988) and Mab 4GA (Cattaneo et al., 1988; Covaceuszach et al., 2009) were conjugated to Biotin and to Acceptor beads, respectively, in both the possible antibody/biotin or antibody/acceptor combinations, following the Perkin Elmer alphaLISA protocol.

The initial results allowed to choose the best configuration: mAb αD11conjugated to acceptor beads and biotinylated mAb 4GA.

The best order of addition and the concentration of the reagents were also assessed:

- 5 μL of the analyte (NGF, proNGF or both),
- 10 μL of the biotinylated mAb 4GA (final concentration 1 mM),
- 10 μL of the mAb αD11conjugated to acceptor beads (final concentration 10 μg/mL),
- First Incubation,
- 25 μL of streptavidin Donor Beads (final concentration 40 μg/mL),
- Second Incubation in the dark.

NGF and proNGF were assayed separately and together (dynamic range: 2000–4 pg/ml, 1:2 dilutions, in duplicates). For each test, three calibration curves were carried out, using purified recombinant proteins as standards: NGF, proNGF, and both neurotrophins in the same well, in the range from 4 to 2000 pg/ml. The dilution were done in AlphaLISA NaCl buffer (Perkin Elmer).

In the first experiments, the first incubation was 60 min, and the second one 30 min. Other assays were carried out, in which the first incubation was 15, 30, or 60 min, the second one 20 min.

The signal was read on the Perkin Elmer instrument EnVision®.

### Is there interference of NGF on proNGF measurement by using the commercial available ELISA Kit?

The CUSABIO Mouse Pro-Nerve Growth Factor (proNGF) ELISA Kit was purchased and tested. Mice brain areas were isolated from 18 months old TgProNGF#72 and WT mice. Two pools of 2 transgenic and 2 WT animals were used and analyzed. The brain tissues were homogenized according to manufacturer's protocol. The total amount of protein in the samples was quantified by Bradford Assay (Sigma-Aldrich). The concentration of total protein measured was 3 mg/ml for both transgenic and WT mice homogenates. The assay was carried out according to manufacturer's instruction.

The samples were homogenized according to the datasheet.

The samples assayed were:

– Serial dilution of TgProNGF#72 and WT brain samples (**Table 5**) in CUSABIO sample buffer,
– Serial dilution of recombinant proNGF (Supplementary Table S2) and NGF in CUSABIO sample buffer,
– Mice tissue spiked with recombinant proNGF (**Table 6**) and NGF,

TgProNGF#72 and WT mice tissues of 18 months were homogenized with the protocol described in Tiveron et al. (2013), and a direct WB were carried out. 150 μg of total protein were loaded onto the gel (criterion, 4–12% Bis-Tris, 12 + 2). The primary antibody used was anti-proNGF (PAb Chemicon Millipore) and the secondary antibody was goat anti-rabbit HRP-conjugated (Jackson).

### Surface plasmon resonance using Biacore

The experiments were all performed with a Biacore 2000 equipment (GE healthcare).

In all the cases, the experiments were performed on CM5 chips with amine coupling. The coupling reaction was performed with the specific kit provided by GE healthcare, according to manufacturer's instructions.

Different antibodies were used: two anti-NGF monoclonal antibodies (the commercial mAb R&D 256 and mAb αD11, Cattaneo et al., 1988); two anti-proNGF antibodies (the commercial mAb Millipore, and mAb FPro10 derived in our laboratory, Paoletti et al., 2012). The antibodies used as ligands were immobilized at a 2000 RU surface concentration of the CM5 chip. The analyte proteins used in all kinetic experiments were injected in PBS (Phosphate Buffer Saline) added with 5% BSA, and at a flow rate of 30 μl/min. The regeneration of the chip was performed with a pulse (10 μl) of 10 mM Glycine pH 1.5. The data analysis was carried out using the BIAevaluation 3.2 Software by Biacore.

For the sake of concentration measurements, report points were placed at the beginning of the dissociation phase, in order to avoid the bulk signal of the buffer.

For the test to reduce the aspecific binding of the biological matrix to the chip, the following conditions were analyzed:

– sample boiling (10 min),
– raising the ionic strength—addition of 250 mM NaCl to the running buffer,
– competition with soluble dextran (Sigma), at a concentration of 0.5 mg/mL in the running buffer or of 10 mg/mL in the samples.

The biological samples analyzed were treated as described for the ELISA assays.

## Rsesults

### Detection of NGF and proNGF from biological sources by immunoprecipitation followed by Western blot: a reference assay

The experiments described in this paper focused on mouse NGF and proNGF, both as recombinant proteins and as proteins derived from mouse brain tissues. In fact, the detection of the neurotrophins remains a more difficult task in this species, with respect to rat, and human samples, where a direct Western blot is possible (Fahnestock et al., 2001).

The recombinant NGF and proNGF were produced and purified as described (Paoletti et al., 2009). As a biological source of NGF and proNGF, for these experiments we used the transgenic mouse TgProNGF#72 and its wild-type counterpart (WT) (Tiveron et al., 2013). This model was chosen because NGF and proNGF are present together, but in larger amounts than in the WT, facilitating their detection.

Immunoprecipitation followed by Western Blot (IP+WB) is a reliable way, albeit of low sensitivity, to detect both NGF and proNGF from biological samples. This approach can be used as a reference, to separately determine the amounts of NGF and proNGF bands in a sample. Samples of different brain areas (Cortex, Basal Forebrain, and Hippocampus) of TgProNGF#72 and WT mice were analyzed by IP+WB, as described (Tiveron et al., 2013). A representative WB, challenged both with anti-proNGF and anti-NGF, is shown in Figure 1A. The amounts of NGF and proNGF, detected in the cortex of the same transgenic mice, are indicated in Figure 1B.

The levels of NGF detected by IP+WB detection of NGF in these samples was compared to those detected by the currently available ELISA assays.

### ProNGF interferes with the determination of NGF by commercial ELISA

Some commercially available ELISA assays were comparatively analyzed in samples containing known amounts of recombinant mouse NGF and proNGF (Paoletti et al., 2009). In the datasheets, Promega Emax and Chemikine declare no cross reactivity with other mature neurotrophins, without mentioning the specificity with respect to proNGF. The possible cross-reactivity with proNGF is reported in the NGF Biosensis datasheet, but is declared to be unimportant, since it is only observed at high protein concentration.

NGF Emax® ImmunoAssay (Promega), one of the most widely used commercial NGF immunoassay, was analyzed first. The assays were carried out using, as primary antibodies, either the Promega anti-NGF mAb, or our own anti-NGF mAb αD11 (Cattaneo et al., 1988). All other steps were carried out according to manufacturer's instruction.

**Figure 2:**
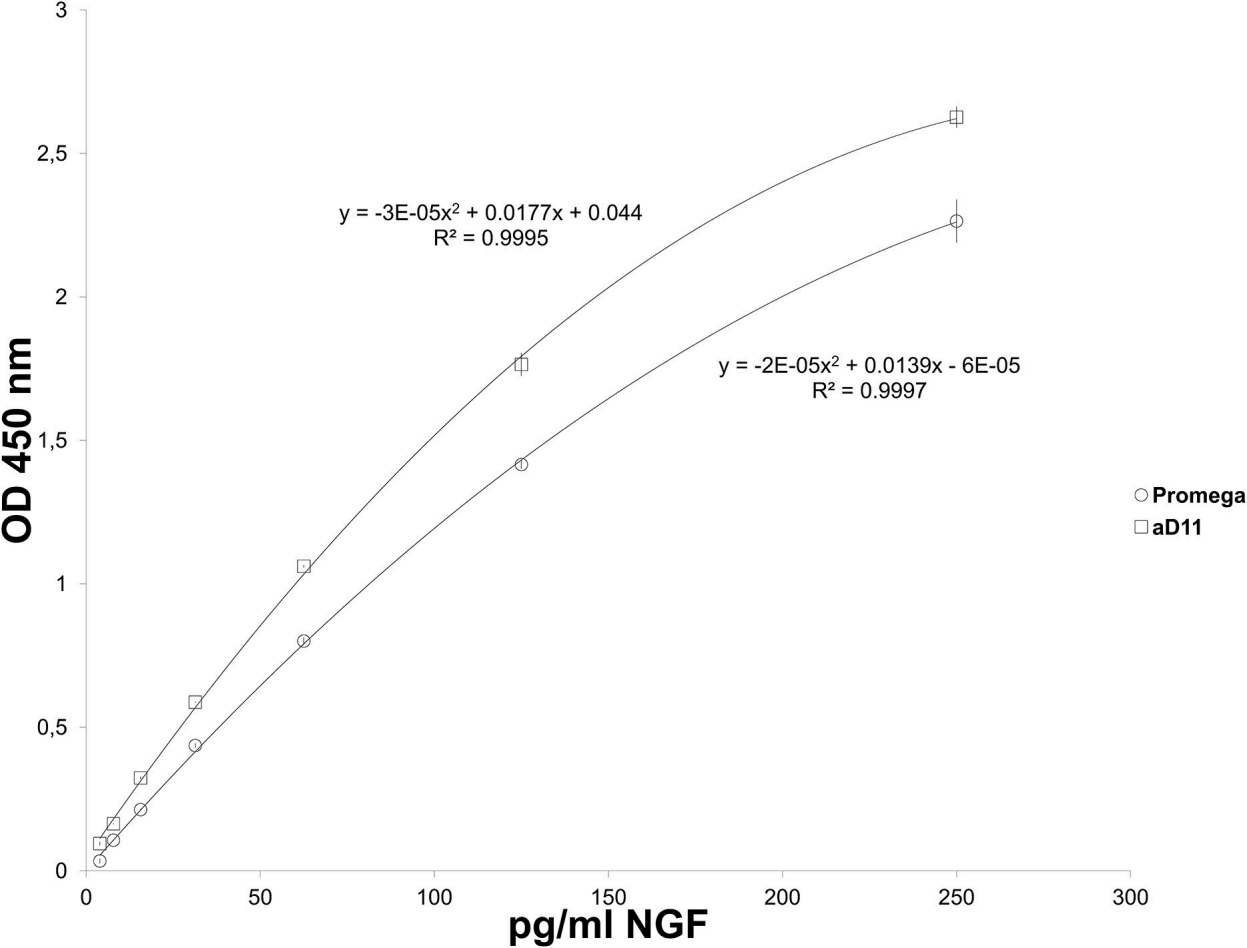
**Emax NGF Promega: NGF Standard curves**. NGF standard curves carried out by using rat mAb Promega (triangles) and mAb αD11 (Cattaneo et al., 1988) (squares) as primary antibody.

Two calibration curves were obtained and interpolated (Figure 2). A curve of proNGF was also performed, in order to verify the extent of cross-reactivity with proNGF. ProNGF was detected by Emax NGF Immunoassay, starting from 1000 pg/ml (Supplementary Table S1). At lower concentrations, proNGF is recognized only by mAb αD11.

**Figure 3:**
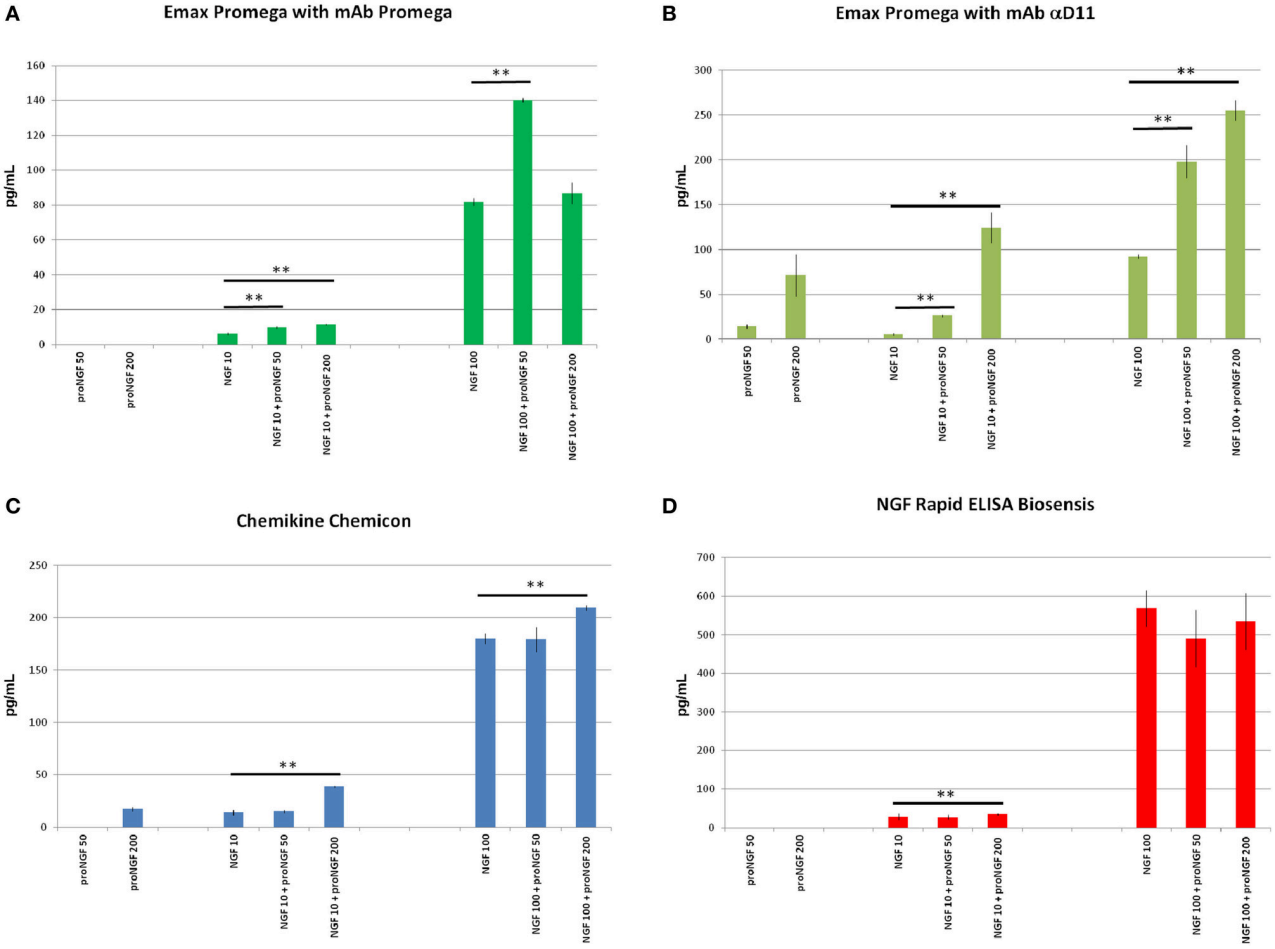
**NGF and proNGF spiked in the sample buffer (supplied by each supplier of the commercial kits)**. The histograms summarize the results of the NGF and proNGF spiked separately and together into the assay buffer of the three different commercial kits for the detection of NGF analyzed. The Promega kit was tested in the two different formats: rat mAb Promega and αD11. Two concentrations of recombinant NGF (10 and 100 pg/ml) and proNGF (50 and 200 pg/ml) were spiked into the assay buffer either alone or together with NGF and measured by using the kits previously described. **(A)** (Emax Promega with mAb Promega), **(B)** (Emax Promega with mAb αD11), **(C)** (Chemikine Chemicon), **(D)** (NGF Rapid ELISA Biosensis) report the values of concentration, interpolated by the calibration curves. The calculated values of the samples spiked with both NGF and proNGF were compared to the spiked value of only NGF. The *t*-student test was carried out and the *p*-values were calculated. Two asterisks on the histograms mean a *p* < 0.001.

Two concentrations of NGF and proNGF were spiked into the assay buffer, either alone or together with NGF, in different stoichiometric ratio (Table 1 and Figure 3). For both antibodies, NGF-spiked samples yielded an acceptable recovery (Table 1 and Figure 3). proNGF spiked-samples were detected, with a lower recovery, only by mAb αD11, thus confirming the results reported in Supplementary Table S1.

Unexpectedly, when proNGF and NGF were spiked together, the interpolated values did not correspond to those obtained for NGF alone. So, despite the fact that the assay did not detect proNGF at these low concentrations, the presence of proNGF altered significantly the measurement of NGF. This interference was not antibody-dependent, since it was observed with both Promega and αD11 anti-NGF mAbs, although αD11 detected proNGF better than mAb Promega.

In order to verify whether this interference was due to the specific layout of the Promega kit, the NGF/proNGF spiking experiments were carried out also with two other commercial kits: ChemiKine NGF Sandwich ELISA (Millipore), and Mouse NGF Rapid ELISA (Biosensis). With both assays, proNGF is poorly detected if tested alone, while NGF alone is largely overestimated, especially by the Biosensis ELISA (Table 1). Remarkably, in both cases, the presence of proNGF markedly interfered with the NGF measurement. Both for the Millipore and Biosensis kit, proNGF altered NGF detection particularly at high spiking concentrations, while the interference is less evident for the 50 pg/ml spiking (Table 1 and Figure 3). In the conditions tested, both kits confirmed what had been observed with the Promega assay.

Thus, in the four different layouts tested, the presence of proNGF altered significantly the measurement of NGF.

The same concentrations of proNGF (50 and 200 pg/ml) were also spiked into diluted brain extracts from TgProNGF#72, either alone or together with NGF (Table 2). The spiked extracts were assayed with the Promega kit, using both Promega and αD11 mAbs, as primary antibodies and the results are reported in Table 2. Curves at different brain extract dilutions were performed and the results obtained with the higher dilution of mouse samples are reported (Table 2).

Surprisingly, all the interpolated values were out of scale. The biochemical analysis on TgProNGF#72 cortex, carried out by IP+WB (Figures 1A,B), demonstrated that the amount of NGF in that sample was about 5 ng/mg of total protein. In the same analysis, proNGF was estimated at 32 ng/mg of total protein. Therefore, in the samples analyzed by ELISA (100 μL of TgProNGF#72 cortex diluted 1:200), the total amount of NGF should be about 70 pg. This estimated NGF value is in the calibration curve concentration range and should be correctly revealed by the assay. On the contrary, the interpolated values were largely out of scale. These results confirmed those obtained with the spikings in assay buffer, leading to the conclusion that the presence of proNGF interferes with the determination of NGF by ELISA.

We conclude that the currently available commercial NGF assays cannot give a reliable measure for NGF, when proNGF is also present in the sample. This aspect cannot be considered negligible, because most natural samples are likely to contain both NGF and proNGF (Fahnestock et al., 2001; Bierl et al., 2005).

### ProNGF interferes with the determination of NGF by *ad hoc* ELISA formats

In the attempt to solve the interference effects described above and develop an ELISA for the differential detection of proNGF and NGF, a number of different *ad hoc* sandwich formats were tested, using both commercial antibodies and antibodies produced in our lab (See Section Methods).

In particular, the specific differential kinetics for NGF and proNGF of the anti-NGF mAb αD11 was exploited. mAb αD11 binds NGF very fast and dissociates very slowly (sub-pM KD) (Paoletti et al., 2009). On the contrary, proNGF is also rapidly bound by mAb αD11, but is released very rapidly, resulting in a nM KD (Paoletti et al., 2009).

Due to these kinetic features of anti-NGF mAb αD11, after a short incubation, NGF should be completely captured by the antibody and remain stably bound, while proNGF should dissociate and be washed away. Table 3 reports all the formats tested, using mAb αD11 as the coating antibody, with a short incubation time. These formats were also affected by interference. The values obtained with proNGF and NGF together were different from the sum of the values of the two neurotrophin forms, assayed separately. In all cases, there was not a well-defined interference trend, since the obtained values depend on the relative input ratio and on the concentrations of the two neurotrophin forms.

**Table 3:** **In house ELISA for the detection of NGF (Exploiting the mAb αD11 fast kinetics)**.

**SCHEME**			
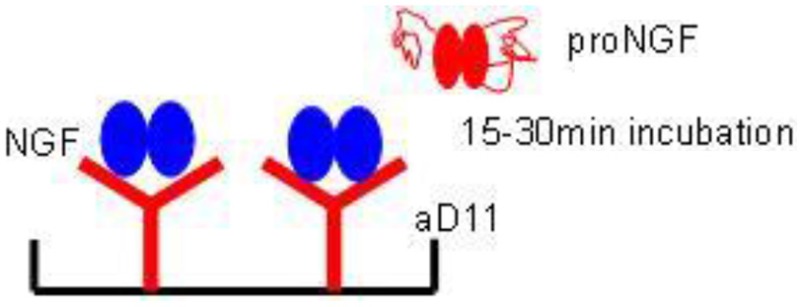			
**Coating**	**Primary**	**Sensitivity**	**Observations**
mAb αD11 M20 Santa Cruz Anti-NGF Sigma Anti-proNGF Sigma Anti-proNGF scFV FPro10	H20 Santa Cruz	ng/ml	Test with recombinant NGF and proNGF, separately and together. Different ratio assayed. The values corresponding to proNGF and NGF together are different from the sum of the values of the neurotrophins, assayed separately. There is not a clear trend, the values depend on the relative ratio of the neurotrophins. The anti-proNGF gave a signal, demonstrating that the proNGF was not completely washed away and interferes with NGF detection.

*ELISA sandwich format based on the kinetic of mAb αD11. After 15–30 min, based on the respective association and dissociation constants, only NGF should remain tightly bound to αD11, while proNGF should be washed out*.

**Figure 4:**
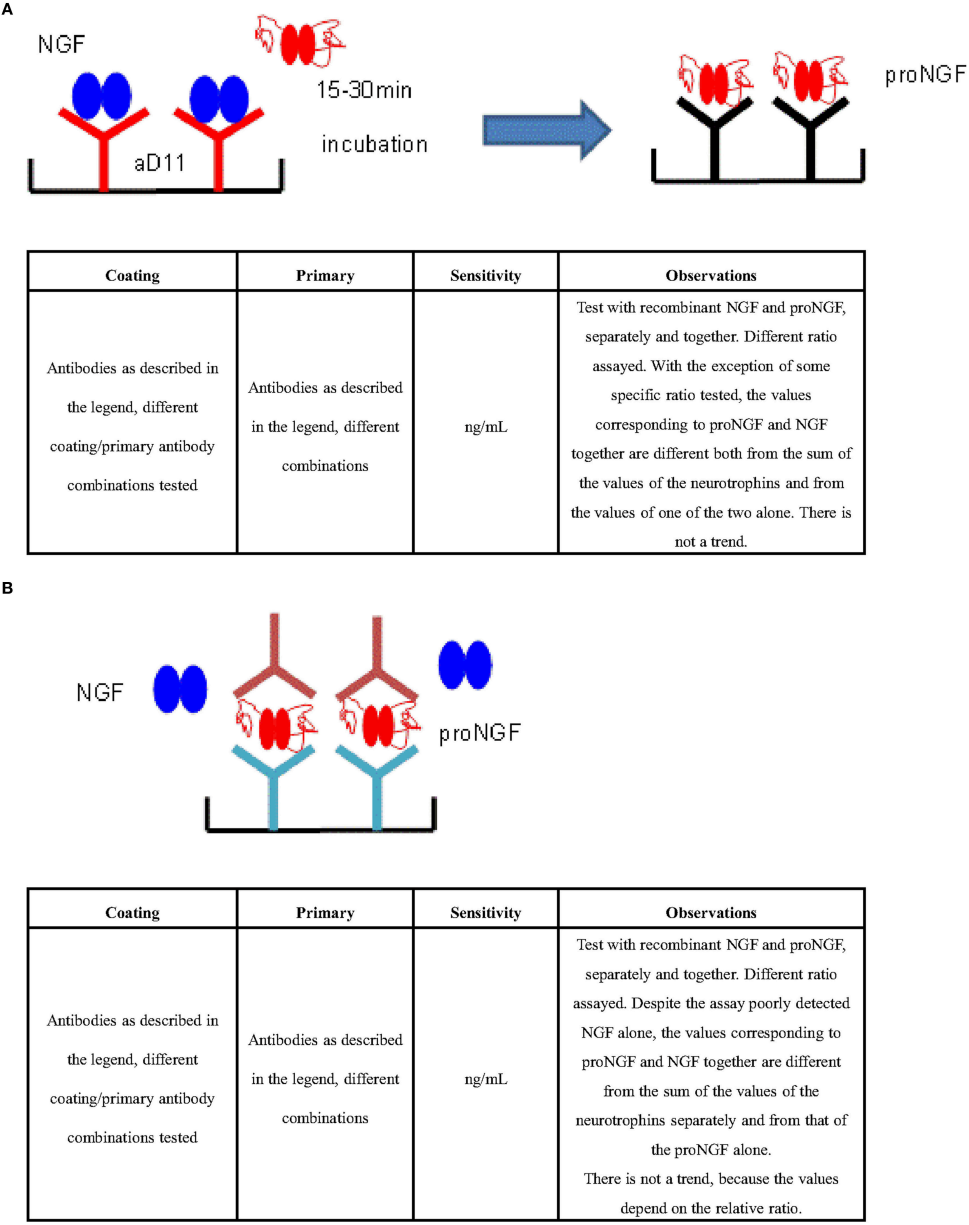
**ELISA for the differential detection of NGF and ProNGF**. **(A)** Strategy: capture NGF and measure proNGF (Exploiting the mAb αD11 fast kinetics). ELISA sandwiches format based on a pre-capturing of NGF by a treatment of the sample with mAb αD11 (Cattaneo et al., 1988) on a solid support. Subsequent detection of proNGF by traditional sandwich ELISA. Antibodies tested in different combinations: Anti-NGF pAb H20 (Santa Cruz no. sc-548), Anti-proNGF scFV FPro10 (Paoletti et al., 2012), Anti-NGF pAb M20 (Santa Cruz no. sc-549), Anti-NGF pAb (Sigma no. N 6655), Anti-proNGF pAb (Sigma no. P 5498), mAb αD11 (Cattaneo et al., 1988), Anti-NGF mAb 256 (R&D no. MAB256). **(B)** Strategy: capture proNGF and measure proNGF (Exploiting anti—proNGF antibodies in ELISA sandwich). Antibodies tested in different combinations: Anti-proNGF scFV FPro10 (Paoletti et al., 2012), mAb αD11 (Cattaneo et al., 1988), Anti-NGF pAb M20 (Santa Cruz no. sc-549), Anti-proNGF Novus (no. S-080-100), Anti-NGF mAb 256 (R&D no. MAB256), Anti-proNGF mAb (clone EP1318Y) (Millipore no. 04-1142), Anti-proNGF pAb Chemicon (Millipore no. AB9040), Anti-proNGF pAb Alomone (no. ANT-005), Anti-NGF Abnova (no. PAB0755).

The interference was also observed with a different assay format, by pre-clearing the sample with an anti-proNGF antibody and measuring free NGF in the flow through (Figure 4). In conclusion, interference between NGF and proNGF appears to be a general phenomenon, independent of the specific formats of the assay.

### ProNGF interferes with the determination of NGF by surface plasmon resonance (SPR)

Surface Plasmon Resonance (Christensen, 1997; Quinn et al., 1997; Frostell-Karlsson et al., 2000; Gillis et al., 2002; Samsonova et al., 2002; Kure et al., 2005; Yang et al., 2005; Thillaivinayagalingam et al., 2007; Mytych et al., 2009) was pursued as an alternative to sandwich ELISA.

CM5 chips with amine coupling chemistry were used in the direct binding format (antibodies as chip-bound ligands and neurotrophins as analytes).

Different anti-NGF and anti-proNGF antibodies were immobilized on the surface. The use of both anti-NGF and anti-proNGF antibodies should make it possible, in principle, to measure NGF and proNGF in a sample in parallel. Different surface densities were tested, to identify the best conditions for the immunoassay (not shown).

**Figure 5:**
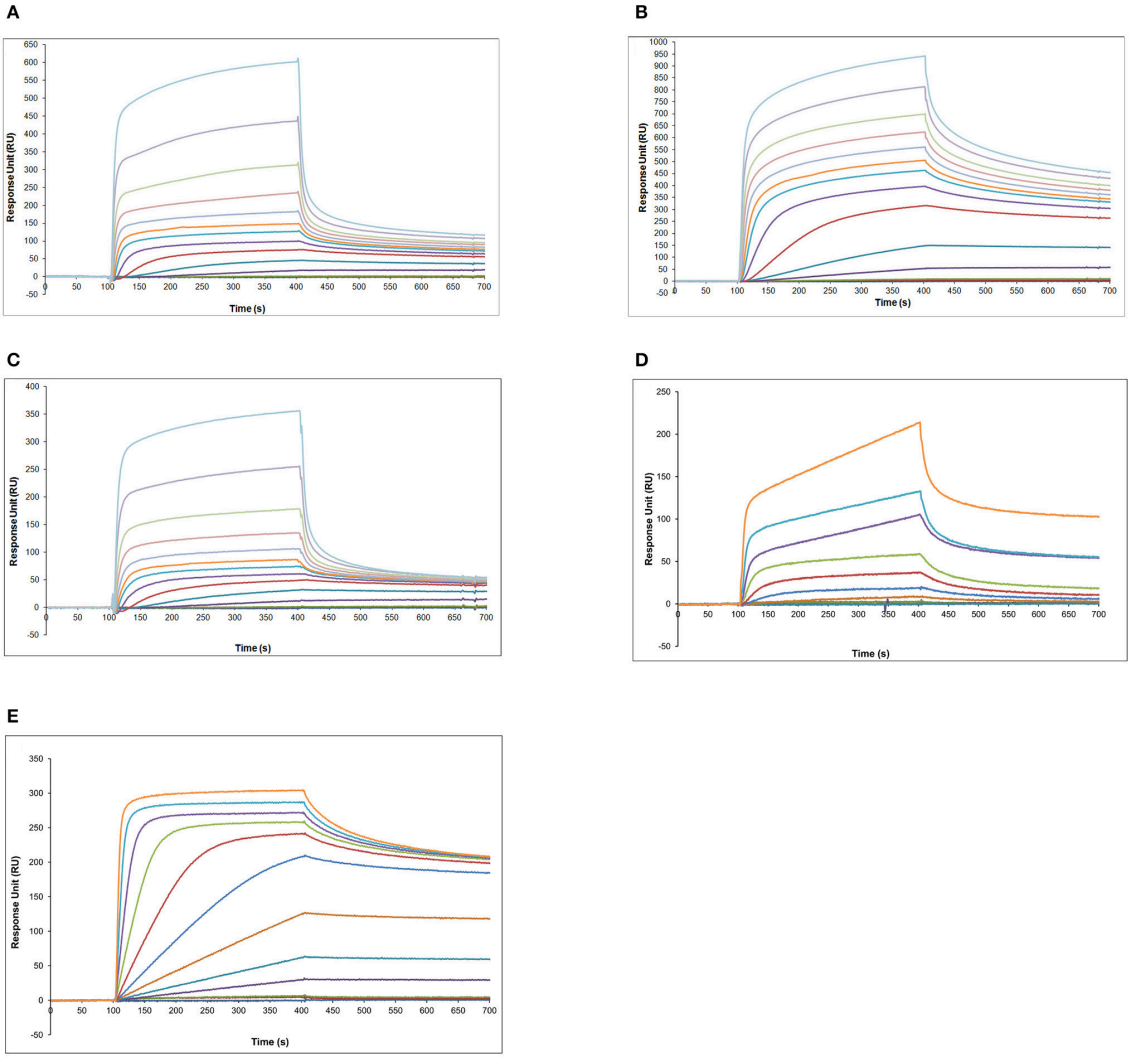
**SPR analysis—calibration curves obtained with proNGF and NGF over the panel of different antibodies. (A)** Different proNGF concentrations tested on anti-proNGF mAb FPro10. Concentrations in nM (from top): 500, 333, 222, 148, 98, 65, 33, 16, 8, 4, 2, 1, 0.5; **(B)** Different proNGF concentrations tested on anti-proNGF mAb Millipore. Concentrations in nM (from top): 500, 333, 222, 148, 98, 65, 33, 16, 8, 4, 2, 1, 0.5; **(C)** Different proNGF concentrations tested on anti-NGF mAb αD11. Concentrations in nM (from top): 500, 333, 222, 148, 98, 65, 33, 16, 8, 4, 2, 1, 0.5; **(D)** Different proNGF concentrations tested on anti-NGF mAb R&D. Concentrations in nM (from top): 100, 50, 25, 12.50, 6.25, 3.2, 1.6, 0.8, 0.4, 0.2, 0.1; **(E)** Different NGF concentrations tested on anti-NGF mAb R&D. Concentrations in nM (from top): 100, 50, 25, 12.50, 6.25, 3.2, 1.6, 0.8, 0.4, 0.2, 0.1.

At first, calibration curves were measured, using recombinant proNGF and NGF flowing over the various antibodies (Figure 5). Well-behaved calibration curves were obtained for the binding of mature NGF to anti-NGF antibodies (Supplementary Figure S1). However, since these antibodies recognize also proNGF (Figures 5C,D), this format cannot be used for the direct detection of mature NGF from a mixture of NGF and proNGF.

As for proNGF, calibration curves could be determined for proNGF with the two anti-proNGF antibodies, with good reproducibility and concentration range (Figures 5A–D, Supplementary Figure S2). The best sensitivity (0.1 nM) was achieved for the Millipore anti-proNGF (Supplementary Figure S2), while it was 0.5 nM for anti-proNGF mAb FPro10.

To increase the sensitivity of the assay and to measure NGF specifically, a number of different sandwich formats, with different antibody combinations, were tested, including the use of Protein L (Svensson et al., 1998) to capture the αD11 anti-NGF antibody. No significant improvement was obtained (data not shown). Therefore, the direct measurement was used for all following experiments.

**Figure 6:**
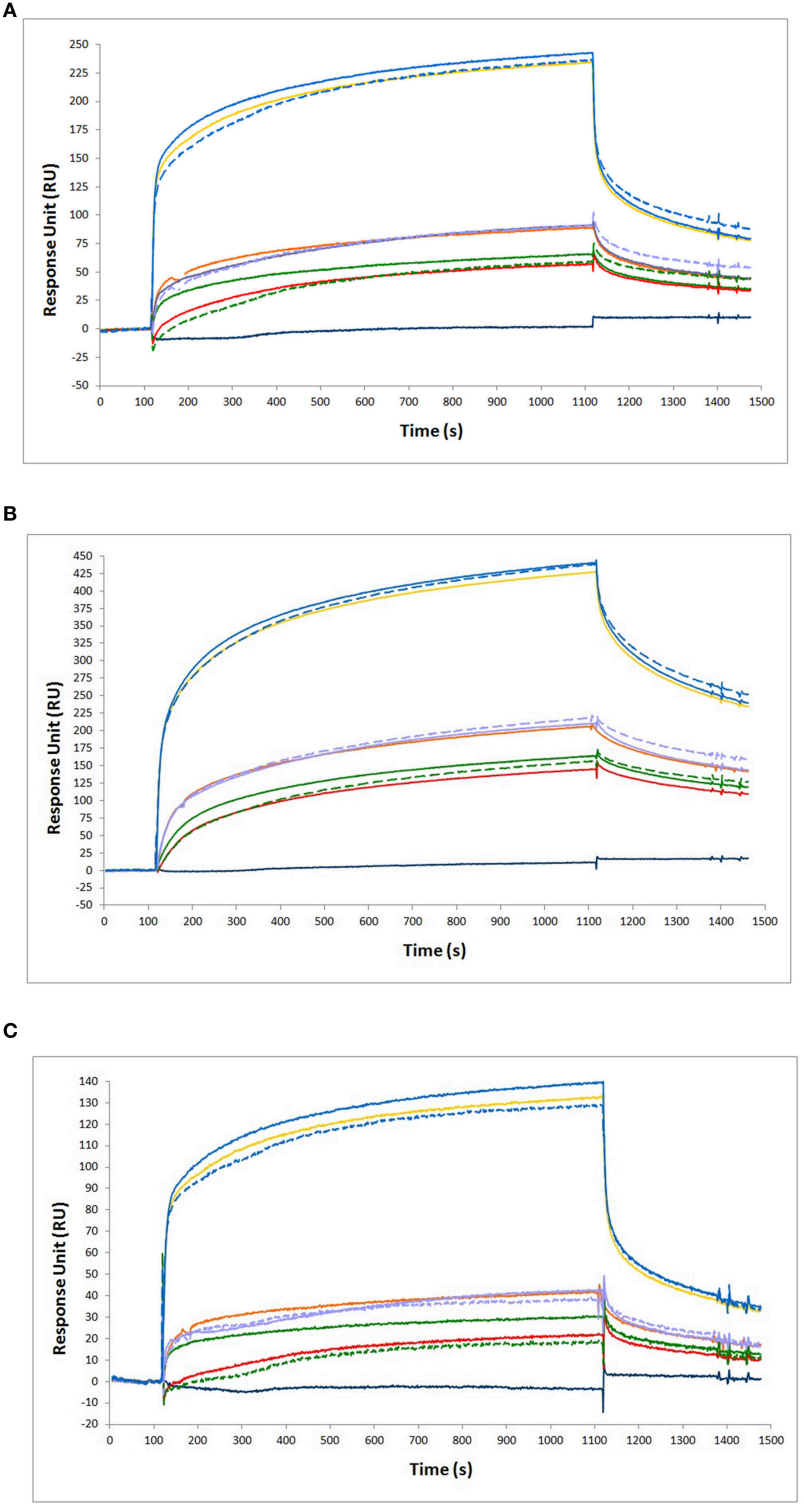
**Binding analysis of NGF and proNGF mixtures to different antibodies**. The following antibodies were immobilized on the chip for SPR experiments. **(A)** anti-proNGF mAb FPro10; **(B)** anti-proNGF mAb Millipore; **(C)** anti-NGF mAb αD11. In all the panels, the curves represent the following analytes from top to bottom: 200 nM proNGF + 20 nM NGF (solid blue line, experimental value); 200 nM proNGF (solid yellow line, experimental value); 200 nM proNGF + 20 nM NGF (segmented blue line, theoretical value); 40 nM proNGF + 20 nM NGF (solid purple line, experimental value); 40 nM proNGF (solid orange line, experimental value); 40 nM proNGF + 20 nM NGF (segmented purple line, theoretical value); 20 nM proNGF + 20 nM NGF (solid green line, experimental value); 20 nM proNGF (solid red line, experimental value); 20 nM proNGF + 20 nM NGF (segmented green line, theoretical value); 20 nM NGF (solid dark blue line, experimental value). The segmented lines represents the theoretical curves for the point-to-point algebraic sum of the experimental curves of the single components, at the concentrations indicated.

A test was made, to verify whether NGF and proNGF, mixed together, interfere with each other, when detected by SPR. As shown in Figure 6, the results obtained for a mixture of NGF and proNGF are not equivalent to the sum of the results obtained for the two proteins alone. The presence of both neurotrophin forms in a sample gives rise to an interference effect. This is detected as a higher response signal (solid lines in Figure 6) compared to the theoretical sum of the experimental curves of the single components (dotted lines of the corresponding color in the Figure 6). This interference effect is variable, depending on the relative ratio of the two proteins in the sample. While for the anti-proNGF antibodies (mAb FPro10 and mAb Millipore) this effect is negligible (Table 4), the interference effect was more significant for the anti-NGF mAb αD11 (Table 4).

**Table 4:** **Calculations of the theoretical value of the measured RU for the single NGF or proNGF components and comparison to the experimental one**.

**Sample**	**Anti-proNGF FPro10 (% of the theoretical value)**	**Anti-proNGF Millipore (% of the theoretical value)**	**Anti-NGF mAb αD11 (% of the theoretical value)**
20 nM proNGF + 20 nM NGF	112	105	166
40 nM proNGF + 20 nM NGF	101	97	111
200 nM proNGF + 20 nM NGF	103	100	108

In conclusion, as for the ELISA, NGF and proNGF mixtures do not behave in a linear way, since the response measured over an anti-NGF antibody is not simply additive. The measurement always overestimates the NGF value in the mixtures, although the percentage of overestimation is variable, depending on the NGF/proNGF ratio. However, it is noticeable that the interference effect, in this case, is significantly lower than that affecting the ELISA assay and has always the same sign. On the other hand, the measures of proNGF with both anti-proNGF antibodies tested in SPR were less affected by the interference of the mixtures and thus gave reliable results also in the case of mixtures. Thus, SPR represents a promising and better starting point to develop an assay for the specific detection of NGF and proNGF from samples in which the two proteins are mixed (See below).

### AlphaLISA for the selective detection of NGF: proof of principle

**Figure 7:**
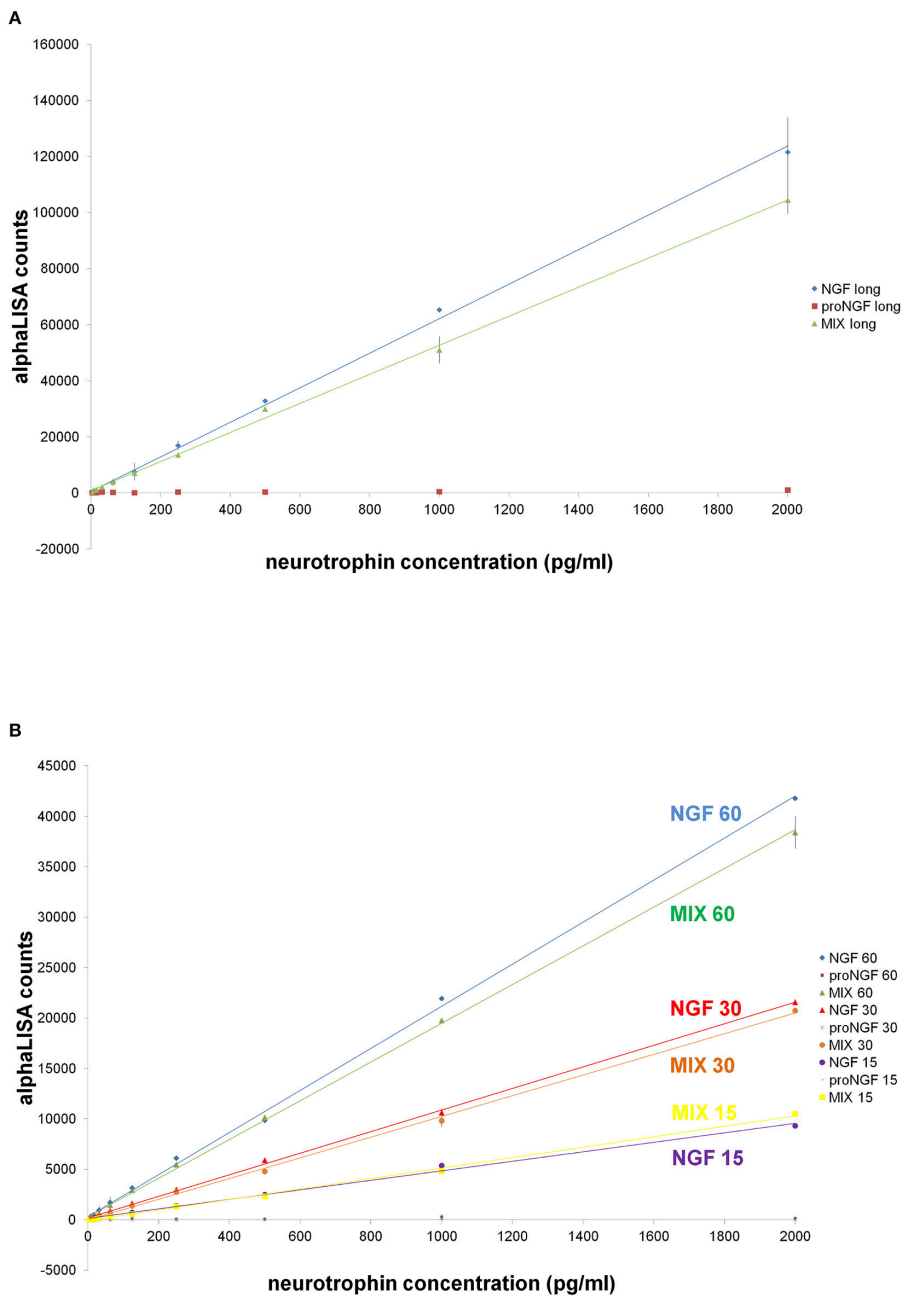
**AlphaLISA experiments: curves of NGF, proNGF, mix of NGF + proNGF, comparison at different incubation times**. Curves of NGF, proNGF, mix of NGF + proNGF, linearly interpolated. NGF and proNGF were assayed separately and together (dynamic range: 2000–4 pg/ml, 1:2 dilutions, in duplicates), in different stoichiometric ratio. The dilution were done in AlphaLISA NaCl buffer (Perkin Elmer). The protocol is described in the methods sections and called for two incubation times. In **(A)** the first incubation time lasted 60 min and the second one 30 min, as suggested by the manufacturer's protocol. In **(B)**, a comparison between different incubation times is shown. The first incubation time was 15, 30, or 60 min, while the second one was 20 min for all the curves. The signal was read on the Perkin Elmer instrument EnVision®.

AlphaLISA is a highly sensitive immunoassay technology (Perkin Elmer) (PerkinElmer, 2008). AlphaLISA experiments were carried out, using the anti-NGF mAb αD11 (Cattaneo et al., 1988) conjugated to acceptor beads and the biotinylated anti-NGF mAb 4GA (Covaceuszach et al., 2009). proNGF was not detected, when assayed alone (Figures 7A,B). With the longer incubation times, also in this case, we observed interference of proNGF on the NGF measurement (Figures 7A,B). However, surprisingly, this interference decreased by shortening the incubation time. Indeed, for the 15 min incubation data, the curves of NGF alone and of the mix of the two neurotrophin forms were almost overlapped (Figure 7B). Thus, with AlphaLISA we have found conditions that minimize the perturbing reciprocal interference of NGF and proNGF.

### Is there interference of NGF on proNGF measurement by using the available commercial ELISA kit?

Similarly to what we did for NGF, we tested the only commercially available proNGF ELISA kit (Mouse proNGF ELISA Kit CUSABIO), in order to assess if there is also an interference of NGF on proNGF measurement. We chose the Mouse proNGF ELISA Kit CUSABIO, because it is the only one in which the datasheet indicates that the assay is applicable to mouse tissue homogenates, despite it does not indicates which ones.

We chose to firstly measure proNGF in the brain of TgProNGF#72 and WT mice in order to validate the assay.

The raw data obtained were good, with low standard deviation between duplicates (Supplementary Figure S3). The measured values did not follow the dilution factor, neither for TgProNGF#72 nor for WT brain tissues (Table 5). Moreover, the interpolated values were similar between transgenic and WT samples, while TgProNGF#72 mice brain contains considerably more proNGF than the WT, as determined by IP+WB. As evident from Supplementary Table S2, the assay did not detect recombinant proNGF. In all the cases tested, the assay did not recognize NGF, as expected (not shown).

**Table 5:** **ProNGF CUSABIO kit: serial dilution of the samples derived from brain extract of TgProNGF#72 and WT mice**.

**Samples**	**Dilution**	**Calculated concentration (pg/ml)**	**Moltiplicated for dilution factor (pg/ml)**	**OD 450 nm ± Standard deviation**
TgproNGF#72	1:100	76.0	7596.2	0.1615 ± 0.006
	1:50	88.1	4407.2	0.1835 ± 0.004
	1:25	98.6	2465.1	0.202 ± 0.004
	1:12.5	111.3	1391.2	0.224 ± 0.008
	1:2	177.6	355.2	0.332 ± 0.007
WT	1:100	89.8	8982.7	0.1865 ± 0.012
	1:50	107.5	5375.7	0.2175 ± 0.002
	1:25	162.3	4057.1	0.308 ± 0.075
	1:12.5	165.8	2072.0	0.3135 ± 0.006
	1:2	266.6	533.2	0.4625 ± 0.071

*The table reports, for each dilution of the samples, the values of concentration of proNGF, simply interpolated, multiplicated for the dilution factor, and the raw data of OD at 450 nm*.

The brain tissue from TgProNGF#72 mice and from the WT, spiked with proNGF, gave results similar to those obtained without spiking (Table 6).

**Table 6:** **proNGF CUSABIO kit: Spiking of recombinant mouse proNGF into TgProNGF#72and WT mice brain samples**.

**Spiking in: (1:100 dilution)**	**Spiking of proNGF (pg/ml)**	**Interpolated value (pg/ml ± Standard deviation)**
TgproNGF#72	750	61.6 ± 0.006
	94	60.8 ± 0.001
	0	76.0 ± 0.006
WT mice	750	78.43 ± 0.001
	94	80.91 ± 0.006
	0	89.8 ± 0.012

*Two concentration (750 and 94 pg/ml) of recombinant proNGF were spiked into TgproNGF#72 and WT mice brain tissues (1:100 dilution). The interpolated values obtained were compared to the non-spiked sample*.

To confirm that the results of the assay were not due to an intrinsic problem of the biological samples used, the same mouse tissues were analyzed by WB. The assay confirmed the presence of proNGF in TgProNGF#72, as expected (data not shown).

We can conclude that this assay is not applicable to brain tissue homogenates. Moreover, it does not detect recombinant proNGF. An explanation for the last issue might be that the datasheet specifies that the curve must be carried out only by using the undefined standard protein supplied with the kit (which we did in parallel).

For these reasons, the interference of proNGF on NGF measurement could not be evaluated for this proNGF ELISA.

### Surface plasmon resonance (SPR) assay for the selective detection of proNGF: proof of principle

The positive results obtained by SPR with anti-proNGF antibodies, showing the absence of interference in mixtures of recombinant NGF and proNGF (Figure 6) prompted us to test SPR for the selective detection of proNGF in biological samples. This is particularly relevant, since use of the only commercially available proNGF ELISA kit (Mouse proNGF ELISA Kit CUSABIO) consistently and convincingly showed that it is not applicable to brain tissue extracts (Tables 5, 6).

**Figure 8:**
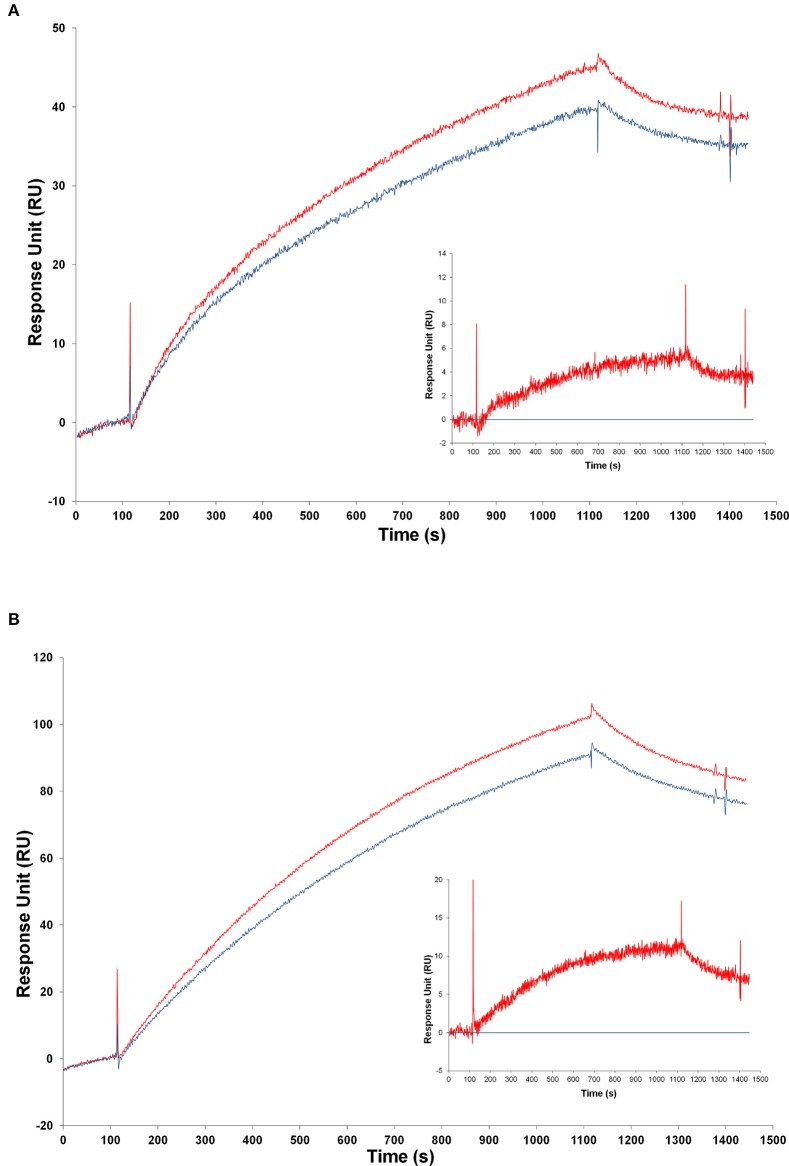
**Analysis of transgenic and wild-type mice samples by SPR**. Injection of samples from transgenic and from WT mice over the FPro10 anti-proNGF antibody **(A)** and over the Millipore anti-proNGF antibody **(B)** (blank subtracted curves). Representation of the SPR curves after subtraction of the WT mice signals. Curve of TgproNGF#72 mice subtracted the curve of WT for the FPro10 anti-proNGF antibody (**A**—insert), and for the Millipore anti-proNGF antibody (**B**—insert). In all panels: Red curve: TgproNGF#72, blue curve: WT.

TgProNGF#72 mice were used as a source of NGF and proNGF in a biologically relevant context. Dilutions of the whole brain homogenates were made, in order to find the lowest possible interference by the sample matrix and avoid the saturation of the system (not shown). A specific signal for proNGF was detected, significantly higher in samples from TgProNGF#72 mice than from WT mice (Figures 8A,B). However, the difference between the signals from the transgenic and WT samples was lower than expected, on the basis of the proNGF levels determined by IP+WB (Figure 1A) (Tiveron et al., 2013). Thus, a very high non-specific binding, to the dextran chip surface, by molecules in the extract different from the neurotrophins, was observed. A number of different conditions were tried, to reduce non-specific binding. Among these: blocking with BSA, heat denaturation of the samples, in order to better expose the epitope, increase of buffer ionic strength, addition of dextran as a soluble competitor, either in the buffer or in the samples. However, none of these conditions gave significant improvements in the non-specific component of the signal (not shown). The only exception was dextran, which slightly improved the results (not shown).

Despite the difficulty in reducing completely the non-specific component of the binding, the difference in proNGF amount in transgenic vs. WT animals could be measured by subtraction of the two curves (Figures 8A,B—inserts). The average calculated concentrations of proNGF in the brain extract, after subtraction of the WT mice signal, from the TgProNGF#72 one, were: 100 nM using FPro10 mAb as detecting antibody; 10 nM using anti-proNGF mAb Millipore. The reference value measured from the same TgProNGF#72 brains, by IP+WB, was 20 nM. Thus, the proNGF amounts calculated by SPR are of the same order of magnitude of those obtained using the IP+WB method, with some differences in the detection by the two proNGF antibodies. These differences could be ascribed to the different epitopes recognized.

These experiments provide a validated proof of principle, for the application of the SPR technique for proNGF measurements with anti-proNGF antibodies on the chip, and show a promising way in future experiments, for the optimization of the assay.

## Discussion

Measuring the relative amounts of NGF and proNGF using sensitive and specific immunoassays would be crucial for diagnostic purposes (Capsoni et al., 2000; Fahnestock et al., 2001; Counts and Mufson, 2005; Capsoni and Cattaneo, 2006; Tiveron et al., 2013; Chen et al., 2014; Iulita and Cuello, 2014; Zhu et al., 2016). While different immunoassays to measure NGF are currently available (See Section Methods), no validated immunoassay for proNGF has been reported so far. A recent report on the measurement of proNGF lacks proper controls in the set-up of the assay (Soligo et al., 2015).

One of the aims of this work was to assess if the currently available methods for measuring NGF are affected by the presence of proNGF in the same sample of NGF, and vice versa. We found, very surprisingly, that the concomitant presence of NGF and proNGF in a sample dramatically alters the measurement of the individual proteins. This interference effect is demonstrated both with recombinant purified NGF and proNGF in buffer and with NGF and proNGF from biological samples. Three different techniques (ELISA, SPR, and alphaLISA) have confirmed the interference, to a different extent for the different methods. Moreover, we set the methodological foundations for new immunoassays for the selective detection of NGF or proNGF, avoiding this interference: alphaLISA for NGF and SPR for proNGF measurements. The results clearly show that by using these methods, and the conditions found, the interference effect becomes negligible.

The results presented here clearly demonstrate that when proNGF is added to NGF, the amount of NGF detected by current immunoassay methods is neither equal to that of NGF alone, nor to the simple sum of the two proteins. Depending on the chosen antibody (Supplementary Table S3), and on the relative concentrations of the two proteins, the measured outcome can be additive or altered in unpredictable directions and thus not possible to be evaluated. In any case, this interference had been hitherto not described. Therefore, caution should be taken when analyzing NGF in a biological sample and when interpreting the large amount of data in the literature. When a quantitative analysis of the two neurotrophins is described, it is important to clarify if the techniques employed are able to distinguish between NGF and proNGF. Moreover, if NGF and proNGF co-exist in the analyzed samples (as most often in biological samples), the absence of interference should be demonstrated with the techniques used, in order for the measurement to be considered reliable. Although we focused in this paper on samples from mouse origin and from recombinant mouse NGF and proNGF, we are quite confident that our observations can also be extended to human samples, since we observed the same kind of behavior with the recombinant human proteins (not shown).

This interference effect was higher in ELISA assay, than in SPR. This could be explained taking into consideration that ELISA technique operates at steady-state, while SPR is measuring in a kinetic mode. Therefore, the kinetic properties of the antibodies could help in preventing significant interfering effects in SPR.

The SPR results clearly show that this technique can be used to develop a valid assay for the measurement of proNGF. Some technical problems due to the high background by interference of unknown components of the biological sample still need to be addressed, before a reliable and sensitive assay can be released, but the presented results show the way forward.

As for the alphaLisa measurements of NGF, the results clearly showed that with a short incubation the interference was virtually abolished, while the interference was higher with longer incubation times. This promising result toward the clean measurement of NGF, in a mixture with proNGF, is most likely due to the differential kinetics properties of mAb αD11 vs. NGF and proNGF (Paoletti et al., 2009). After a short incubation, NGF was captured by mAb αD11 and remained stably bound, while proNGF was not revealed. The same strategy that failed in the classical ELISA format (See above), worked, instead, in the alphaLISA set up. In this case, mAb αD11 was not fixed to a solid support, as it was in the ELISA setting, and therefore was not at limiting concentrations, but at a concentration high enough to quantitatively capture NGF. In these conditions, the fast kinetic avoided the binding of proNGF. The very promising alphaLISA approach should be now tested and validated for the measurement of NGF in biological samples.

All the results indicated that the coexistence of proNGF and NGF in the same sample alters the correct selective measurement of one of the two molecules. This issue, on one hand, complicates the development of a good assay, but, on the other hand, opens important and interesting biological questions. Is there a molecular cross-talk between precursor and mature neurotrophin forms, possibly involving the formation of NGF/proNGF supramolecular structures, similar to the recently described NGF dimer of dimer? (Covaceuszach et al., 2015). Does the nature of this oligomeric cross-talk depend on NGF/proNGF ratio? The answer to these questions arises from the interesting interplay between the components of the system and careful investigations will be required to give a definitive answer. Finally, it could be interesting to investigate whether this interference is typical of the NGF/proNGF system or if it could involve also other pro-neurotrophins and their mature forms, for which there is also a need to develop fully validated assays.

## Author contributions

AC, FM, and FP conceived the experiments. FM and FP performed the experiments and analyzed the data. AC, FM, and FP wrote the manuscript, prepared the figures, and reviewed the manuscript.

## Funding

FISM Research Project 2013/R/6. MIUR, project PRIN #2010N8PBAA_006. European Community's Seventh Framework Program PAINCAGE Grant number 603191.

### Conflict of interest statement

The authors declare that the research was conducted in the absence of any commercial or financial relationships that could be construed as a potential conflict of interest.
