# Supplementary material for: NGF and proNGF Reciprocal Interference in Immunoassays: Open Questions, Criticalities, and Ways Forward
Source: Front Mol Neurosci. 2016 Aug 3;9:63. doi: 10.3389/fnmol.2016.00063 (PMC4971159; doi:10.3389/fnmol.2016.00063)
Supplement: Supplementary file 1 [file DataSheet1.PDF]

Supplementary Material

NGF and proNGF reciprocal interference in immunoassays : open questions, criticalities and ways forward

Francesca Malerba[1,2¶], Francesca Paoletti[1,2¶], Antonino Cattaneo[1,2,*]

[1] Neurotrophic Factors and Neurodegenerative Diseases Unit, European Brain Research Institute, "Rita Levi-Montalcini" Foundation, Rome, Italy

[2] BioSNS Laboratory, Scuola Normale Superiore, Pisa, Italy

*Corresponding Author:

e-mail: antonino.cattaneo@sns.it (AC)

[¶] these authors contributed equally to this work

Supplementary Figure S1 - SPR analysis - calibration curves obtained with proNGF and NGF over the panel of different antibodies.

Panels: **A** – Different proNGF concentrations tested on anti-proNGF mAb FPro10. Concentrations in nM: 500, 333, 222, 148, 98, 65, 33, 16, 8, 4, 2, 1, 0.5; **B** – Different proNGF concentrations tested on anti-proNGF mAb Millipore. Concentrations in nM: 500, 333, 222, 148, 98, 65, 33, 16, 8, 4, 2, 1, 0.5; **C** – Different proNGF concentrations tested on anti-NGF mAb αD11. Concentrations in nM: 500, 333, 222, 148, 98, 65, 33, 16, 8, 4, 2, 1, 0.5; **D** – Different proNGF concentrations tested on anti-NGF mAb R&D. Concentrations in nM: 100, 50, 25, 12.50, 6.25, 3.2, 1.6, 0.8, 0.4, 0.2, 0.1;

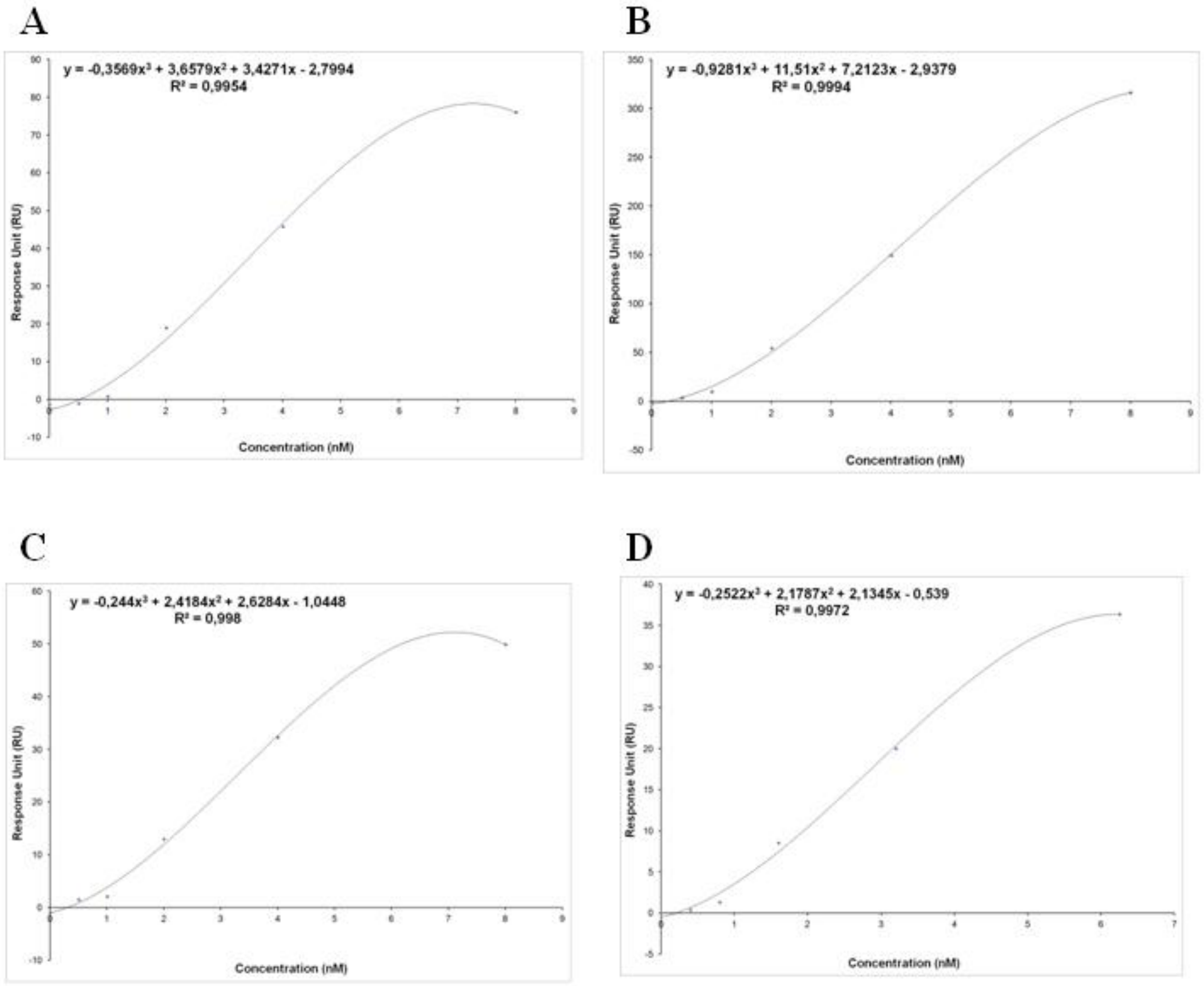

Supplementary Figure S2: SPR analysis - calibration curves obtained with proNGF and NGF over anti-proNGF antibody by Millipore

Panel A - Different proNGF concentrations tested on anti-proNGF mAb Millipore. Concentrations in nM (from top to bottom): 5, 2.5, 1.7, 1.1, 0.74, 0.49, 0.22, 0.14, 0.09, 0.06, 0.

Panel B - calibration curve obtained for the corresponding set of curves

A

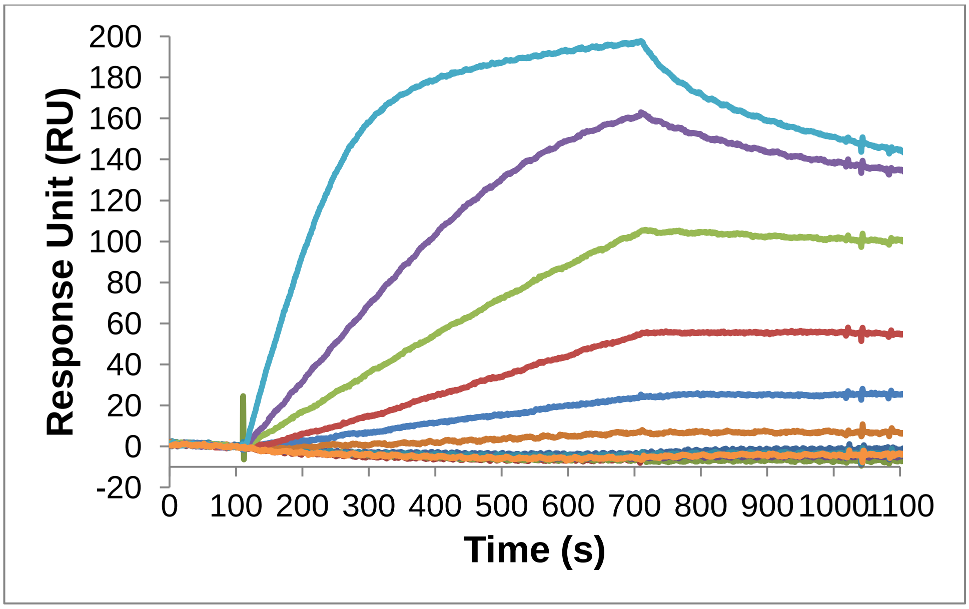

B

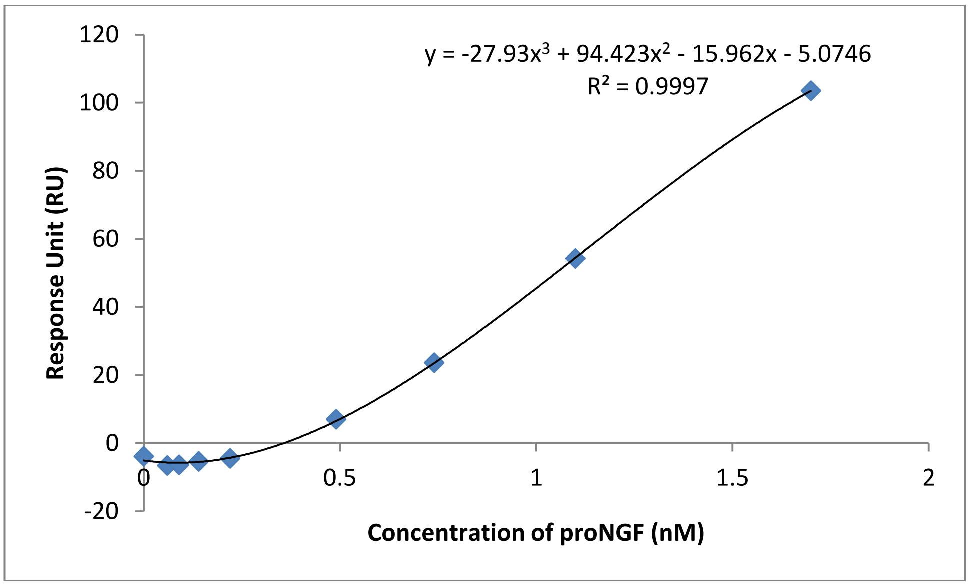

Supplementary Figure S3 - ELISA mouse proNGF Cusabio: standard curve.

proNGF standard curves carried out following the manufacturer's instructions of CUSABIO ELISA test.

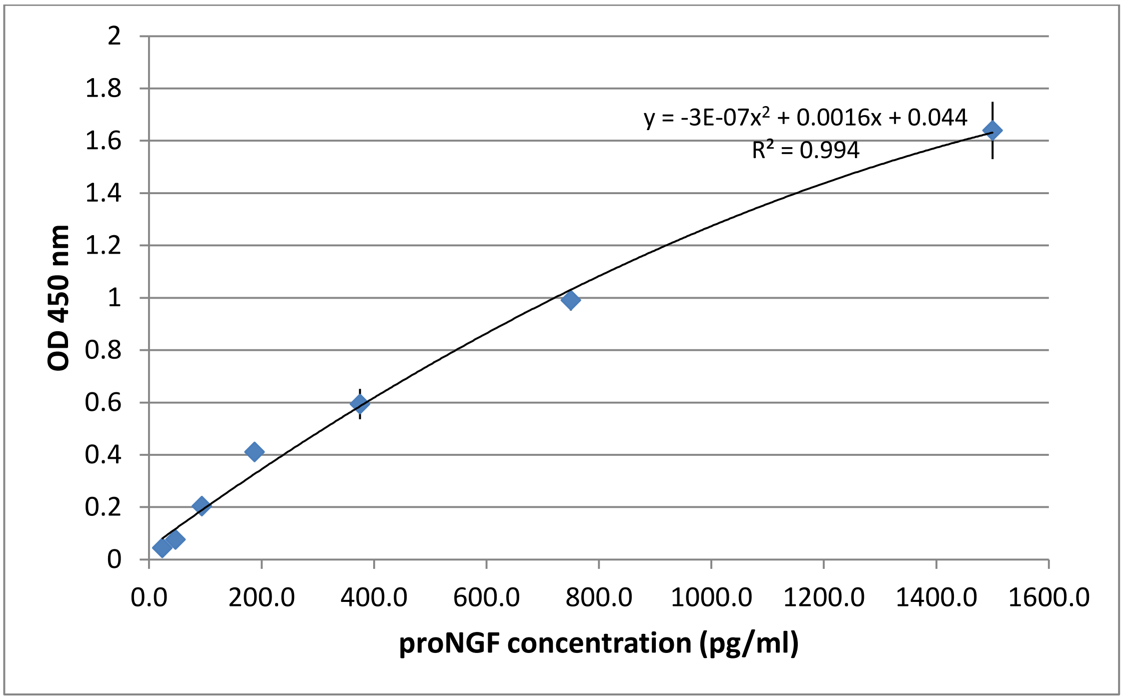

Supplementary Table S1

Emax NGF Promega : proNGF Curve

proNGF concentration	OD 450 nm ± Standard Deviation	
	**Promega**	**mAb αD11**
1 mg/ml	Out of scale	Out of scale
100 ng/ml	Out of scale	Out of scale
10 ng/ml	Out of scale	Out of scale
1000 pg/ml	0.629±0.001	2.185±0.067
100 pg/ml	0±0.006	0.233±0.005
10 pg/ml	0±0.001	0.035±0.028

Curve of recombinant proNGF, in the range from 10 pg/ml to 1mg/ml, assayed in Emax NGF Promega kit, using both mAb αD11 and mAb Promega as primary antibody. The experiment was carried out in triplicate.

Supplementary Table S2

proNGF CUSABIO kit: Standard curve of recombinant mouse proNGF

proNGF concentration (pg/ml)	Interpolated value (pg/ml)	OD 450 nm measured ± Standard deviation
750	6.3	0.0245 ± 0.011
375	1.4	0.014 ± 0.001
187.5	0.7	0.0125 ± 0.001
93.75	-4.3	0.0015 ± 0.005

Interpolated values and measured OD450 nm for recombinant proNGF, produced in our lab, and tested in proNGF CUSABIO kit.

Supplementary Table S3

Summary of the epitopes of the antibodies used in the paper.

Antibody name	Antibody epitope	Immunogen	Reference	Cat. No.
Anti-NGF mAb αD11	NGF Loops I & II (res. 25-32 & 42-46)		Covaceuszach et al, JMB	-
Anti-NGF mAb 256		Mouse myeloma cell line NS0-derived recombinant human beta - NGF Ser122-Ala241 Accession # CAA36832		Anti-NGF R&D no. MAB256
Anti-NGF pAb M20	At the N-terminus of NGF mature chain of mouse origin			Anti-NGF Santa Cruz no. sc-549
Anti-NGF pAb H20	At the N-terminus of the mature chain of NGF of human origin			Anti-NGF Santa Cruz no. sc-548
Anti-NGF pAb Sigma		NGF-2.5S from male mouse submaxillary glands.		Sigma no. N6655
Anti-proNGF scFv FPro10	Residues 74-83 in the pro-peptide of proNGF		Paoletti et al, ABB, 2012	-
Anti-proNGF pAb Sigma		Peptide corresponding to amino acid residues 84-104 of rat NGF (precursor). The sequence is identical in mouse and 16/21 residues identical in human.		Sigma no. P5498
Anti-proNGF mAb Millipore (clone EP1318Y)	N-terminus	Synthetic peptide corresponding to residues near the N-terminus of human NGF-β precursor (pro-NGF).		Anti-proNGF Millipore no. 04-1142

1. Tiveron C, Fasulo L, Capsoni S, Malerba F, Marinelli S, Paoletti F, Piccinin S, Scardigli R, Amato G, Brandi R, Capelli P, D'Aguanno S, Florenzano F, La Regina F, Lecci A, Manca A, Meli G, Pistillo L, Berretta N, Nistico R, Pavone F, Cattaneo A. ProNGF\NGF imbalance triggers learning and memory deficits, neurodegeneration and spontaneous epileptic-like discharges in transgenic mice. Cell Death Differ2013 Aug;20(8):1017-30.
2. Cattaneo A, Rapposelli B, Calissano P. Three distinct types of monoclonal antibodies after long-term immunization of rats with mouse nerve growth factor. J Neurochem1988 Apr;50(4):1003-10.
